# Unveiling barriers to reproductive health awareness among rural adolescents: a systematic review

**DOI:** 10.3389/frph.2024.1444111

**Published:** 2024-11-19

**Authors:** Sri Wahyuningsih, Sri Widati, Sarva Mangala Praveena, Mohammad Wavy Azkiya

**Affiliations:** ^1^ Doctoral Program of Public Health, Faculty of Public Health, Universitas Airlangga, Surabaya, Indonesia; ^2^ Faculty of Public Health, Universitas Airlangga, Surabaya, Indonesia; ^3^ Department of Environmental and Occupational Health, Faculty of Medicine and Health Sciences, Universiti Putra Malaysia, UPM Serdang, Selangor Darul Ehsan, Malaysia; ^4^ Faculty of Nursing, Universitas Jember, Jember, Indonesia

**Keywords:** adolescent, rural, reproductive health, barriers, awareness, knowledge, social norms

## Abstract

**Introduction:**

Sexual and reproductive health (SRH) among adolescents is a critical aspect of global health. Rural adolescents often encounter significant barriers to reproductive health awareness, elevating their risks for unintended pregnancies, sexually transmitted infections (STIs), and other reproductive health issues. This systematic review seeks to identify and analyze the barriers hindering reproductive health awareness among rural adolescents.

**Methods:**

This review followed PRISMA guidelines. Literature searches were conducted in PubMed, ScienceDirect, Google Scholar, and Taylor & Francis, focusing on studies published from 2019 to 2024. Keywords included “Adolescent,” “Rural,” “Reproductive Health,” “Awareness,” and “Barriers.” Studies were screened based on eligibility criteria, and data were extracted and analyzed to identify key barriers at the individual, interpersonal, social/community, and health services levels.

**Results:**

Out of 669 records, 20 studies met the inclusion criteria. Identified barriers at the individual level included lack of knowledge, myths, misconceptions, and feelings of shame and fear. Interpersonal barriers were related to poor communication between parents and adolescents and misinformation from peers. Social and community barriers encompassed rigid social norms, stigma, and discrimination. Health services barriers included limited access and negative experiences with reproductive health services.

**Discussion:**

Rural adolescents face complex barriers to reproductive health awareness driven by factors at the individual, interpersonal, social, and health services levels. Comprehensive interventions, such as educational campaigns, training for healthcare providers, and improved access via mobile or online platforms, are essential to enhance reproductive health awareness and outcomes.

**Systematic Review Registration:**

https://www.crd.york.ac.uk/, PROSPERO (CRD42024554439).

## 1 Introduction

Adolescent reproductive health was included in the global health and development agenda at the International Conference on Population and Development (ICPD) in 1994. Reproductive health is defined as a state of physical, mental, emotional, and social well-being related to the reproductive system (1). Adolescence is a period characterised by the drive to explore sexual activities, yet the lack of knowledge about reproductive health often increases the risk of various reproductive health issues, including unintended pregnancies, abortions, and sexually transmitted infections such as HIV/AIDS (2). Awareness of adolescent reproductive health still shows significant disparities between rural and urban areas (3).

Reproductive health issues among adolescents are significant worldwide. In 2022, the United Nations International Children's Emergency Fund (UNICEF) estimated that 1.65 million adolescents were living with HIV/AIDS. Approximately 1.1 million or 85 percent were in developing countries in Sub-Saharan Africa, with the remainder in Asia and the Americas (4). Additionally, the World Health Organization (WHO) estimated that in 2019 there were 12 million cases of unintended adolescent pregnancies in developing countries (5). The prevalence of sexually transmitted infections significantly increased from 1994–2017 (6). Other studies indicate that disparities in rural areas lead to limited access to information and reproductive health services, contributing to higher health risks among rural adolescents (7–10). The lack of healthcare facilities, increasing service costs, and access difficulties faced by rural communities are major barriers to improving understanding and access to reproductive health services (11). Furthermore, the understanding of social norms in rural areas can influence adolescents' behaviour in making decisions about their reproductive health (12). Highlighting these issues, many barriers still prevent rural adolescents from obtaining information and improving their knowledge related to reproductive health, thereby hindering the development of reproductive health awareness (13).

Although some published systematic reviews provide insights into the factors affecting access to adolescent reproductive health services, such as structural barriers (negative attitudes of healthcare providers, lack of skills, stigma, cost, lack of access to services, privacy concerns) and individual barriers (lack of knowledge), none specifically address the barriers faced by rural adolescents in becoming aware of the importance of reproductive health (14, 15). Therefore, this systematic review aims to better illustrate the obstacles experienced by adolescents in rural areas.

The preparation of this systematic review follows the Preferred Reporting Items for Systematic Reviews and Meta-Analyses (PRISMA) guidelines (16). The PRISMA 2020 guidelines outline several stages in the process, including: defining eligibility criteria, identifying sources of information, selecting data, collecting data, and extracting data. The data obtained will be illustrated using a flow diagram in accordance with the guidelines (17). The use of the PRISMA method will make this systematic review more transparent, comprehensive, and accurate (16).

By identifying and understanding the inhibiting factors affecting reproductive health awareness among rural adolescents, it is hoped that this review can inform the development of more effective and relevant interventions. Therefore, the aim of this study is to elucidate the specific factors that hinder reproductive health awareness among rural adolescents, with the ultimate goal of improving their well-being and reproductive health.

## 2 Methods

**Figure 1:**
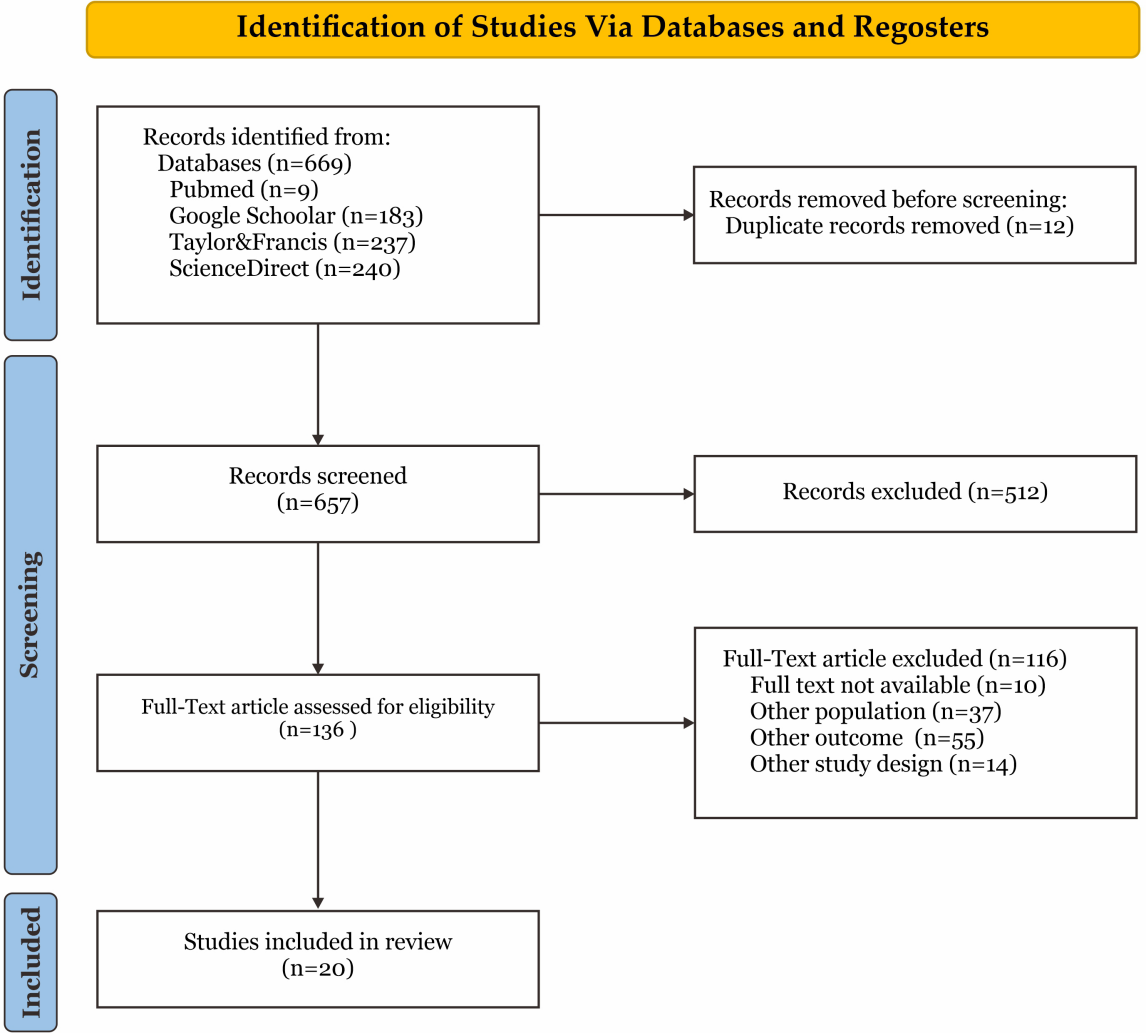
PRISMA flowchart.

The systematic review followed the standards of the “Preferred Reporting Items for Systematic Reviews and Meta-Analyses” (PRISMA). PRISMA criteria were used to identify and screen scientific papers, as illustrated in Figure 1 of the PRISMA flowchart (18).

### 2.1 Identification and selection of studies

The literature search was conducted on 11 May 2024 across four databases: PubMed, ScienceDirect, Google Scholar, and Taylor & Francis. Keywords used included “Adolescent”, “Rural”, “Reproductive Health”, “Awareness”, and “Barriers” employing Boolean operators (AND/OR). The publication range covered the past five years. The search strategy was tailored to each database, as detailed in Supplementary Table S1. A secondary search was performed by manually reviewing the reference lists of the articles included in this review to identify potentially relevant studies.

The eligibility criteria were articulated according to the Population, Exposure, Outcomes, and Study (PEOS) framework for the research question. Specifically, “P” pertained to adolescents residing in rural areas; “E” encompassed barriers to reproductive health awareness; “O” focused on awareness levels regarding reproductive health; and “S” denoted studies with both qualitative and quantitative designs, published within the past five years in peer-reviewed journals in English.

The results of the database searches were imported into Mendeley software, where duplicate studies were identified and subsequently excluded. Titles and abstracts of the studies were independently evaluated based on the eligibility criteria. Following this stage, the studies available online were assessed to ascertain their inclusion status.

### 2.2 Data extraction

Following the PRISMA standards for screening and selection, we retrieved the essential data, including title, author, year, journal, country, study design, population, research aims, and outcome were all used as descriptive information.

### 2.3 Assessment of study quality

In this systematic review, the quality assessment of included articles utilized the Joanna Briggs Institute (JBI) Critical Appraisal Checklist for Quantitative Studies and the JBI Critical Appraisal Checklist for Qualitative Research (19). The former evaluated aspects such as sampling strategy and statistical analysis, while the latter assessed research design and data analysis methods (20). Each checklist facilitated a thorough evaluation of methodological quality and risk of bias, informing the interpretation of study findings (19).

### 2.4 Ethical considerations

This systematic review did not require ethical approval. The authors of the articles reviewed in this systematic review had obtained consent from their research subjects. However, this review was registered under PROSPERO (CRD42024554439).

## 3 Results

A total of 669 records were retrieved from the literature search across four databases. Following the removal of 12 duplicates, 521 records were excluded based on title and abstract screening, resulting in 136 studies for full-text assessment. Subsequently, 123 studies deemed irrelevant were excluded, leaving 20 studies eligible for inclusion in the review (Figure 1). These studies were published between 2019 and 2024, predominantly emerging after 2022, and were conducted in 10 countries, with a focus on the African Continent. A summary of the included studies is shown in Supplementary Table S2.

The findings of the review identified various barriers to reproductive health awareness among rural adolescents from multiple perspectives, including those of adolescents themselves, parents/caregivers, and healthcare providers. The majority of studies (76.9%) explored barriers from the perspective of adolescents (21–33), while a smaller proportion investigated the viewpoints of parents/caregivers (15.3%) (34, 35), and healthcare providers (7.6%) (36).

**Figure 2:**
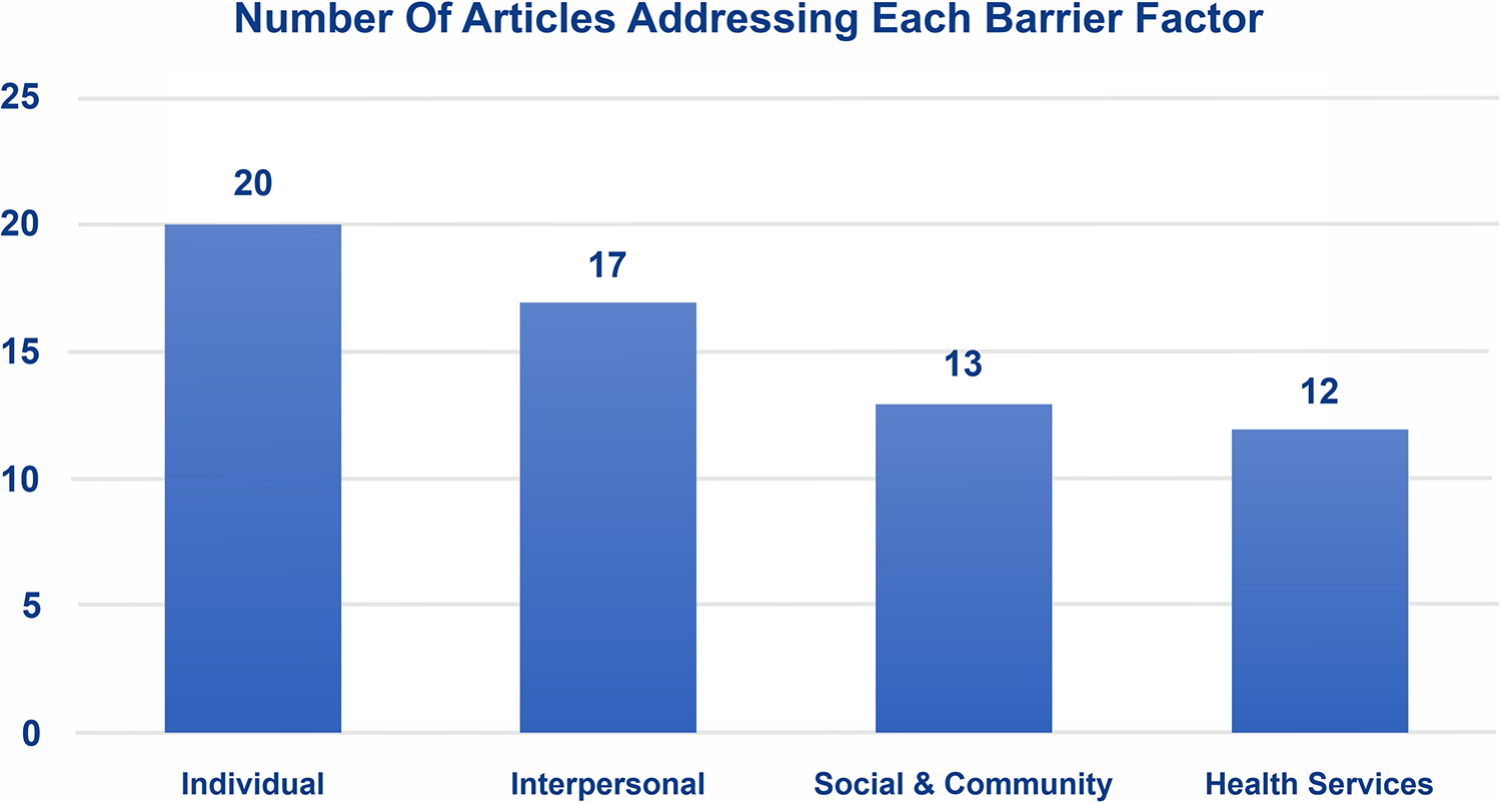
Number of articles addressing each barrier factor.

**Figure 3:**
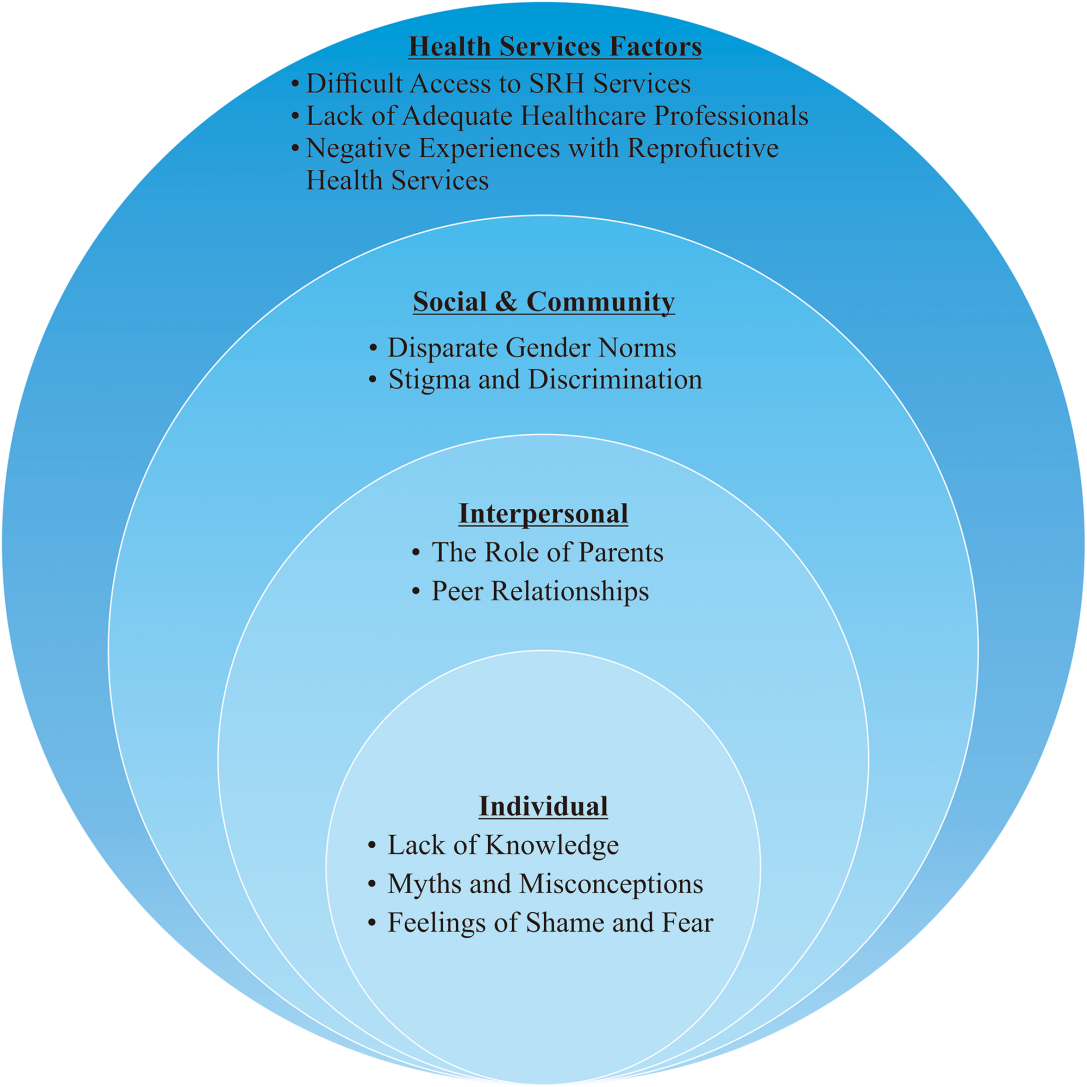
Barriers to reproductive health awareness among rural adolescents.

In this review, we identify factors based on the individual level, interpersonal level, social and community level, and health services level (Figure 3). Our findings reveal that 20 studies highlight individual factors (100%), 18 studies highlight interpersonal factors (90%), 13 studies focus on social and community factors (65%), and 12 studies highlight health services factors (60%) (Figure 2).

### 3.1 Individual factor

Individual factors constitute the most significant barriers to SRH awareness among rural adolescents. These factors include a lack of knowledge, myths and misconceptions, and feelings of shame and fear (21–30, 32–35, 37–42).

#### 3.1.1 Lack of knowledge

Ten studies highlight the low levels of SRH knowledge among rural adolescents (21, 22, 26–29, 34, 36, 38). Knowledge about puberty, menstruation, contraceptives, sexually transmitted infections, and reproductive health in general is typically low among rural populations (24, 39, 42). Many adolescents do not understand the basic biological aspects and the importance of maintaining reproductive health. Rural adolescents sometimes receive incorrect information or hold misconceptions about reproductive health due to prevailing social norms in rural areas (28, 34, 37). Additionally, many rural adolescents are unaware of the existence of reproductive health services in their regions, resulting in a lack of understanding of the benefits of utilising these services (21, 22).

#### 3.1.2 Myths and misconceptions

Six studies mention the emergence of myths and misconceptions among rural adolescents (23, 30, 32, 33, 40, 42). Among rural adolescents, there are various incorrect assumptions about pregnancy, contraception, sexually transmitted infections, menstruation, and reproductive health due to the influence of social and cultural norms. Rural adolescents often believe that first-time sexual intercourse does not cause pregnancy, sexually transmitted infections are only transmitted by sex workers or shared needles, contraception is unnecessary for occasional sexual activity, and discussing sexual issues or seeking sexual health information is considered immoral (23, 30, 32, 33, 40, 42).

#### 3.1.3 Feelings of shame and fear

Feelings of shame and fear are one of the inhibiting factors in reproductive health awareness among rural adolescents. There are seven studies that discuss the issue of feelings of shame and fear as an obstacle in the reproductive health awareness of rural adolescents (24, 28, 34, 35, 37–39). Shame and fear are the result of social and cultural norms that exist in rural communities related to reproductive issues that are still considered taboo or inappropriate to talk about (28, 34, 37, 39). This assumption is also reinforced by the existence of stigma and social punishment when adolescents try to find information related to SRH or when visiting reproductive health services (35). As a result, adolescents become reluctant to seek information and choose to follow existing norms so that awareness related to reproductive health is hampered. This barrier is exacerbated by the lack of family support which further inhibits open communication about reproductive health (34, 38, 39).

### 3.2 Interpersonal factor

In this review, interpersonal factors are also significantly discussed in the literature reviewed. The interpersonal factors focus on two aspects: the role of parents and peer relationships.

#### 3.2.1 The role of parents

The role of parents is a significant barrier to improving reproductive health awareness among rural adolescents for several reasons. In this review, ten studies discuss the role of parents as a barrier to SRH awareness among rural adolescents (21, 22, 26, 28, 29, 34, 35, 39, 40, 42). The lack of communication between parents and children regarding reproductive health topics is often due to social and cultural norms that consider discussions about sexuality to be taboo or inappropriate (28, 39, 40, 42). Parents often feel uncomfortable or lack sufficient knowledge to discuss these topics, resulting in adolescents not receiving the necessary information from reliable sources (21, 26, 29, 35). Additionally, parents' negative attitudes towards sexual education, believing that discussing reproductive health may encourage undesirable sexual behaviour, further exacerbate the situation (39). These barriers create an environment where adolescents are undereducated about reproductive health and do not know how to protect themselves effectively.

#### 3.2.2 Peer relationships

Peer relationships play an important role in shaping adolescent awareness of reproductive health. Peers can be an obstacle or a driver for the creation of awareness related to reproductive health of rural adolescents. This can occur because peer groups consist of individuals of the same age group, so adolescents often find it easier to exchange ideas or information without any restrictions related to reproductive issues (28, 43). On the other hand, adolescents sometimes listen to their peers more than their parents so that parents will have difficulty in providing understanding related to reproductive health. Peer relationships sometimes put pressure to follow the behaviour of their friends in order to be considered the same (34). In some findings, peer relationships that tend to be negative sometimes have risky behaviours and misinformation related to reproductive health (25, 26). As a result, adolescents' awareness of reproductive health is hampered. Conversely, positive peer relationships can increase knowledge and awareness of reproductive health in rural adolescents (25, 33).

### 3.3 Social and community factors

Social and community factors play a highly significant role as barriers to reproductive health awareness among rural adolescents. Social and community factors include social and cultural norms, stigma and discrimination.

#### 3.3.1 Disparate gender norms

Disparate gender norms are still often found in rural areas. Disparate gender norms create a view of men as breadwinners and women taking care of the household (30, 34, 42). As a result, girls' roles are limited and intended to keep them at home so that their parents can supervise them and prepare them to be good wives. This is very different from teenage boys who have more freedom (34). One of the phenomena that occurs due to this perspective is the limited opportunity for adolescent girls to get formal education. In rural areas, education related to reproductive health is often provided at school (38). Other findings related to disparate gender norms show that adolescent girls when married can experience a sense of isolation due to living with their spouse. This change in lifestyle greatly affects adolescent girls' decision-making to seek information or services related to reproductive health (30). The perspective of disparate gender norms that exist in rural areas can be an obstacle for adolescents to have reproductive health awareness, especially adolescent girls.

#### 3.3.2 Stigma and discrimination

Seven studies discuss stigma and discrimination as barriers to adolescents' SRH awareness (25, 29, 32, 35, 40–42). Social stigma that considers discussions about reproductive health to be taboo makes adolescents feel ashamed and afraid to seek information or access reproductive health services, fearing they will be judged or labelled as “naughty” (32, 42). Discrimination from healthcare providers and community members, particularly against adolescent girls and adolescents with disabilities, worsens the situation by preventing them from obtaining the necessary services (29, 35, 40). This discrimination leads to adolescents feeling unsupported and reluctant to seek help, resulting in a lack of knowledge and awareness about reproductive health. Efforts to address these barriers should focus on reducing stigma and discrimination and improving access to adolescent-friendly reproductive health services (25, 41).

### 3.4 Health services factors

#### 3.4.1 Difficult access to SRH services

Difficult access to reproductive health services is due to the lack of adequate healthcare facilities and the long distances to healthcare centres (21, 23, 29, 39–41). In a study conducted in Oyo State, Nigeria, adolescents found it challenging to access quality services (21). In Rwanda and Ethiopia, a lack of healthcare facilities and adequate healthcare professionals led to low utilisation of services (29, 39, 40, 43). Strict lockdowns during the COVID-19 pandemic and fear of COVID-19 transmission have also hindered rural adolescents' access to reproductive health services (41). In addition, health workers are more focused on pandemic response, so reproductive health services are disrupted. These barriers can interfere with the delivery of appropriate information related to reproductive health from health workers to rural adolescents, so that reproductive health awareness becomes minimal (24).

#### 3.4.2 Lack of adequate healthcare professionals

Several literature reviews highlight the shortage of trained healthcare professionals and support for them in providing reproductive health services, resulting in adolescents not receiving the education and services they need (34, 36, 40, 41, 43). Studies in Zambia and Ethiopia found that healthcare providers and community workers did not have adequate training to support reproductive health services (37, 40). Another study during the COVID-19 pandemic in KwaZulu-Natal, South Africa, noted that the lack of healthcare professionals worsened the situation regarding access to reproductive health services (41).

#### 3.4.3 Negative experiences with reproductive health services

Negative experiences with reproductive health services discourage adolescents from seeking help and reduce their awareness and utilisation of these services (22, 24, 25, 35). In a study conducted in Oyo State, Nigeria, adolescents reported unfriendly services (22). Another study in Bangladesh found that adolescents with disabilities faced discrimination (35). In West Bengal, it was found that services were not tailored to adolescents' needs, and negative experiences with healthcare providers deterred adolescents from seeking help (24, 25).

## 4 Discussion

Our findings indicate that the biggest barriers for adolescents are individual factors. The most dominant individual factor is the lack of knowledge related to sexual and reproductive health among rural adolescents. As they grow and develop, adolescents are likely to face pressures to explore sexual activities. However, the lack of knowledge about reproductive health increases the risk of various reproductive health issues, including unintended pregnancies, abortions, and sexually transmitted infections such as HIV/AIDS (2). Gender disparities are evident in high-risk sexual behaviours. Multiple sexual partners, inconsistent use of contraception, and premarital sex are more prevalent among male adolescents than females (44). Female adolescents often become victims of abuse, unintended pregnancies, and cultural stigma, which further threaten their reproductive health (45). Despite widespread knowledge about HIV/AIDS among adolescents, broader aspects of reproductive health remain poorly understood. Adolescents lack knowledge about menstruation, puberty processes, contraception, other sexually transmitted infections, pregnancy, abortion, how to care for reproductive organs, and how to access reproductive health services (46, 47). Generally, adolescents receive reproductive health information at school. This topic is usually included in the school curriculum but is often not taught because teachers feel uncomfortable discussing it (48). However, adolescents who drop out of school do not have access to this information, making them a more vulnerable and less supervised group (49).

Embedded gender norm disparities among adolescents can influence their health behaviours (50, 51). Disparities such as the experience of menstruation in female adolescents have impacts on behaviour, self-confidence, and decision-making, leading to feelings of shame and discomfort (52). These limitations in knowledge and feelings affect SRH awareness among rural adolescents.

This study also found that interpersonal factors, such as the role of parents and peers, play a significant role in adolescents' awareness of SRH. Most rural adolescents still rely on their families, with decision-making often handled by parents (28). Additionally, parental decision-making is influenced by social norms that view adolescents, particularly girls, as lacking control and having their opinions undervalued (53). Poor communication between adolescents and parents regarding reproductive health issues results in adolescents being less capable of making informed decisions (54). Even though parents are preferred sources of information about reproductive health and are aware of their role in adolescent reproductive health, a lack of confidence and prevailing religious norms create an uncomfortable communication environment between parents and adolescents. Alternatives for adolescents to obtain information include teachers, peers, and siblings (55, 56). Peer-based education can be an effective intervention in improving reproductive health knowledge and behaviour among adolescents. Peer-based education is a structured intervention where adolescents are trained to disseminate accurate reproductive health information to their peers, as opposed to negative influence or peer pressure. These interventions consistently show improvements in SRH knowledge, such as knowledge about HIV, contraceptive use, and sexually transmitted infections (STIs) (57). The effectiveness of peer education depends on several conditions, including structural support, comprehensive educational materials, and supervision by trained health professionals (58).

Another barrier is the community and social factors, which significantly affect SRH awareness among rural adolescents. Disparate gender norms, still frequently encountered in rural areas, and the perception of SRH as a taboo by various groups are influenced by the religious and cultural perspectives of different ethnicities (59). In some belief systems, matters related to sexuality are considered private and unnecessary to discuss, with the belief that discussing them may lead to negative outcomes (60). Unmarried adolescents or women who visit SRH services face stigmatisation due to the perception that anyone seeking SRH services must be sexually active (61). This perception stems from social norms that view sexually active adolescents or unmarried individuals as immoral (62). Various interventions are necessary to address issues within the community and social domains. Community-based approaches involving religious leaders, teachers, parents, and community leaders are crucial in overcoming these social barriers (63). In some communities, sexual and reproductive health education programs in schools are very effective without having to override local cultural values (64). This will not only reduce stigma but also increase public acceptance of SRH services.

Another important factor to consider is health services. This study highlights that health services can also be a barrier. Most rural adolescents receive SRH education at school. Although SRH topics are comprehensively covered in schools, some adolescents who drop out do not receive this information (10). In several countries, rural communities often face disparities in accessing healthcare services. Rural adolescents still experience difficulties in accessing reproductive health services, inadequate healthcare, and increasing costs, especially in remote areas (65, 66). Although reproductive health is a right for everyone, racial-ethnic disparities in accessing adequate reproductive healthcare still exist in multiracial-ethnic countries (67, 68). Additionally, discrimination against individuals with disabilities is still found in healthcare facilities, in terms of physical accessibility, attitudes, and communication barriers (69).

The importance of SRH knowledge is significantly influenced by personal, community, cultural, religious, policy, and regulatory factors. These factors overlap and interact with each other, resulting in impeded awareness of the importance of implementing SRH (61). Evidence-based interventions are crucial to address these overlapping factors (70). Awareness campaigns on reproductive health for parents and adolescents, mobile/online-based outreach, peer educator training, and school-based education can be implemented to enhance reproductive health awareness (71, 72).

Although this study has employed a comprehensive approach and adhered to PRISMA guidelines throughout the review process, we acknowledge that it still has limitations. The literature search was restricted to publications written in English, which may have overlooked relevant studies written in other languages on the subject. The review predominantly includes studies from the African continent, which may limit the generalizability of the findings to other regions. Additionally, the literature search was confined to studies published between 2019 and 2024, potentially excluding older relevant studies that could provide further insights. Another limitation of this study is the variation in study design, population, and context included, which results in data heterogeneity and may limit the uniformity of conclusions.

## 5 Conclusion

This systematic review reveals that the primary barriers to reproductive health awareness among rural adolescents include a lack of knowledge, myths and misconceptions, and feelings of shame and fear driven by social and cultural norms. Interpersonal barriers such as poor communication between parents and adolescents, and misinformation from peers exacerbate these issues. Furthermore, rigid social norms, stigma, discrimination, and limited access to reproductive health services further prevent adolescents from obtaining necessary information and services. Comprehensive interventions, including educational campaigns, training for service providers, and improved accessibility through mobile or online platforms, are essential to enhance reproductive health awareness and outcomes among rural adolescents.

## Data Availability

The original contributions presented in the study are included in the article/Supplementary Material, further inquiries can be directed to the corresponding author.
