# Corrigendum: Unveiling barriers to reproductive health awareness among rural adolescents: a systematic review

**DOI:** 10.3389/frph.2024.1537155

**Published:** 2024-12-11

**Authors:** Sri Wahyuningsih, Sri Widati, Sarva Mangala Praveena, Mohammad Wavy Azkiya

**Affiliations:** ^1^ Doctoral Program of Public Health, Faculty of Public Health, Universitas Airlangga, Surabaya, Indonesia; ^2^ Faculty of Public Health, Universitas Airlangga, Surabaya, Indonesia; ^3^ Department of Environmental and Occupational Health, Faculty of Medicine and Health Sciences, Universiti Putra Malaysia, UPM Serdang, Selangor Darul Ehsan, Malaysia; ^4^ Faculty of Nursing, Universitas Jember, Jember, Indonesia

**Keywords:** adolescent, rural, reproductive health, barriers, awareness, knowledge, social norms

A Corrigendum on Unveiling barriers to reproductive health awareness among rural adolescents: a systematic review By Wahyuningsih S, Widati S, Praveena SM and Azkiya MW (2024). Front. Reprod. Health. 6:1444111. doi: 10.3389/frph.2024.1444111

Incorrect Funding

In the published article, there was an error in the Funding statement. The original statement incorrectly mentioned “The author(s) declare financial support was received for the research, authorship, and/or publication of this article. This study was funded by the Indonesian Education Scholarship [Beasiswa Pendidikan Indonesia (BPI)], provided by the Ministry of Education, Culture, Research and Technology of the Republic of Indonesia. Recipient: Sri Wahyuningsih, Indonesian Education Scholarship Identification Number: 202327091301”. The correct Funding statement appears below.

FUNDING

The author(s) declare financial support was received for the research, authorship, and/or publication of this article. This study was funded by the Higher Education Funding Center (Balai Pembiayaan Pendidikan Tinggi, BPPT) and the Indonesia Endowment Fund for Education (Lembaga Pengelola Dana Pendidikan, LPDP), under the Ministry of Education, Culture, Research and Technology of the Republic of Indonesia. Recipient: Sri Wahyuningsih, Indonesian Education Scholarship Identification Number: 202327091301.

The authors apologize for this error and state that this does not change the scientific conclusions of the article in any way. The original article has been updated.

