# Supplementary material for: Unveiling barriers to reproductive health awareness among rural adolescents: a systematic review
Source: Front Reprod Health. 2024 Nov 19;6:1444111. doi: 10.3389/frph.2024.1444111 (PMC11611845; doi:10.3389/frph.2024.1444111)
Supplement: Supplementary file 1 [file Table1.docx]

**Table 1. Search query May 11, 2024**

| Pubmed | ((((((((adolescent[MeSH Terms] ) OR (teenager[MeSH Terms])) OR (youth[MeSH Terms])) AND (rural population[MeSH Terms])) OR (rural health service[MeSH Terms])) OR (rural health[MeSH Terms])) AND (((((((reproductive health[MeSH Terms]) OR (sexual health[MeSH Terms])) OR (sex education[MeSH Terms])) OR (contraception[MeSH Terms])) OR (family planning services[MeSH Terms])) OR (sexually transmitted disease[MeSH Terms])) OR (hiv infections[MeSH Terms]))) AND (((awareness[MeSH Terms]) OR (health knowledge, attitudes, practice[MeSH Terms])) OR (health education[MeSH Terms]))) AND (((((barrier[MeSH Terms]) OR (obstacle[MeSH Terms])) OR (inhibition psychology[MeSH Terms])) OR (health services accessibility[MeSH Terms])) OR (social norms[MeSH Terms])) |
| --- | --- |
| Sciencedirect | Adolescents AND Rural Population AND Reproductive Health AND Awareness OR Health Knowledge OR Health Education AND Barriers |
| Taylor&francis | [[All: adolescent] OR [All: teenager] OR [[All: youth] AND [All: rural]]] AND [[All: population] OR [All: rural]] AND [All: health] AND [[All: service] OR [All: rural]] AND [All: health] AND [All: reproductive] AND [[All: health] OR [All: sexual]] AND [[All: health] OR [All: sex]] AND [[All: education] OR [All: contraception] OR [All: family]] AND [All: planning] AND [[All: services] OR [All: sexually]] AND [All: transmitted] AND [[All: diseases] OR [All: hiv]] AND [All: infections] AND [[All: awareness] OR [All: health]] AND [All: knowledge, attitudes,] AND [[All: practice] OR [All: health]] AND [All: education] AND [[All: barrier] OR [All: obstacle] OR [All: inhibition]] AND [[All: psychology] OR [All: health]] AND [All: services] AND [[All: accessibility] OR [All: social]] AND [All: norms] AND [Publication Date: (01/01/2019 TO 12/31/2024)] |
| Google Schoolar | Adolescents OR Teenagers OR Youth AND Rural Population OR Rural Health Services OR Rural Health AND Reproductive Health OR Sexual Health OR Sex Education OR Contraception OR Family Planning Services OR Sexually Transmitted Diseases OR HIV Infections AND Awareness OR Health Knowledge OR Health Education AND Barriers OR Obstacles OR Health Services Accessibility OR Social Norms |
