# Supplementary material for: Unveiling barriers to reproductive health awareness among rural adolescents: a systematic review
Source: Front Reprod Health. 2024 Nov 19;6:1444111. doi: 10.3389/frph.2024.1444111 (PMC11611845; doi:10.3389/frph.2024.1444111)
Supplement: Supplementary file 2 [file Table2.docx]

**Table 2. Summary of the studies included in the systematic review**

| **Author (year) Country** | **Title** | **Partisipant** | **Main Results (barriers)** |
| --- | --- | --- | --- |
| **Qualitative (n = 12)** | | | |
| Tiwari et al. (2022), Nepal [28] | “Our mothers do not tell us”: a qualitative study of adolescent girls’ perspectives on sexual and reproductive | 22 adolescent girls | Myths and Misconceptions, The Role of Parents, Social and Cultural Norms |
| Chilambe et al. (2023), Zambia [36] | Experiences of teachers and community-based health workers in addressing adolescents’ sexual reproductive health and rights problems in rural health systems: a case of the RISE project in Zambia | 21 in-depth interviews with teachers and community based health workers | Lack of Knowledge, Lack of Adequate Healthcare Professionals |
| Achen et al. (2022), South-Western Uganda [34] | Gendered Experiences of Parent–Child Communication on Sexual and Reproductive Health Issues: A Qualitative Study Employing Community-Based Participatory Methods among Primary Caregivers and Community Stakeholders in Rural South-Western Uganda | 19 female and 18 male primary caregivers who had adolescent children aged 10–14 years | Myths and Misconceptions, Feelings of Shame and Fear, The Role of Parents, Lack of Adequate Healthcare Professionals |
| Reddy et al., (2022) Telanggana, Indonesia [27] | Knowledge, attitude, and practices related to reproductive and sexual health among adolescent girls in a rural community of Telangana | 324 adolescent aged 10–19 years | Lack of Knowledge, Myths and Misconceptions |
| Akatukwasa et al., (2023), Uganda [38] | Narratives of most significant change to explore experiences of caregivers in a caregiver-young adolescent sexual and reproductive health communication intervention in rural south-western Uganda | 30 participants | Myths and Misconceptions, Peer Relationships |
| Mukandagano et al., (2022), Rwanda [29] | Reproductive Health Knowledge and Services Utilization among Rural Adolescents in Rwamagana District, Rwanda | 392 adolescent aged 15–19 years | Lack of Knowledge, The Role of Parents, Stigma and Discrimination, Difficult Access to SRH Services |
| McGuire et al., (2024), Peru [30] | Seeking information and services associated with reproductive health among rural Peruvian young adults: exploratory qualitative research from Amazonas, Peru | 24 participants | Lack of Knowledge, Feelings of Shame and Fear |
| Chimwaza-Manda et al., (2024) [33] | Sexual health knowledge acquisition processes among very young adolescent girls in rural Malawi: Implications for sexual and reproductive health programs | 43 participants | Myths and Misconceptions, Peer Relationships, Social and Cultural Norms |
| Natsayi et al. (2022) South Africa [41] | The sexual and reproductive health needs of school-going young people in the context of COVID-19 in rural KwaZulu-Natal, South Africa | 69 participants | Lack of Knowledge, Stigma and Discrimination, Difficult Access to SRH Services, Lack of Adequate Healthcare Professionals |
| Power et al., (2023), Bangladesh [35] | The Sexual and Reproductive Health of Adolescents with Cerebral Palsy in Rural Bangladesh: A Qualitative Analysis | 24 adolescents with CP and 76 caregivers | The Role of Parents, Stigma and Discrimination, Negative Experiences with Reproductive Health Services |
| Rasweth & Nisha (2022), India [42] | The unspoken plight of married adolescent girls in rural Tamil Nadu: Narrative summary on unmet sexual and reproductive health needs and barriers | 14 young women participated | Lack of Knowledge, Feelings of Shame and Fear, The Role of Parents, Social and Cultural Norms, Stigma and Discrimination |
| Zuma et al. (2020), KwaZulu-Natal, South Africa [25] | Young people’s experiences of sexual and reproductive health interventions in rural KwaZulu-Natal, South Africa | 137 participants young people | Lack of Knowledge, Feelings of Shame and Fear, Peer Relationships, Social and Cultural Norms, Stigma and Discrimination, Negative Experiences with Reproductive Health Services |
| **Mix-method (n = 1)** | | | |
| Banerjee et al., (2023) Bengal [24] | Utilisation of adolescent reproductive and sexual health services in a rural area of West Bengal: A mixed-method study | 326 adolescent aged 15–19 years | Lack of Knowledge, Feelings of Shame and Fear, Peer Relationships, Negative Experiences with Reproductive Health Services |
| **Quantitave (n = 7)** | | | |
| Ilori et al., (2020) Oyo State [21] | Awareness and Utilization of Adolescent Reproductive Health Services Among In-School Adolescents in Urban and Rural Communities in Oyo State | A total of 225 rural and 225 urban respondents participated in this study | Lack of Knowledge, The Role of Parents, Peer Relationships, Social and Cultural Norms, Difficult Access to SRH Services |
| Ilori et al., (2024), Oyo State [22] | Expectations and Experiences of Urban and Rural In-School Adolescents of Adolescent Reproductive Health Services in Oyo State, Nigeria | A total of 226 rural and 226 urban respondents participated in this study | Lack of Knowledge, The Role of Parents, Peer Relationships, Social and Cultural Norms, Difficult Access to SRH Services, Negative Experiences with Reproductive Health Services |
| Adjie et al., (2022), Indonesia [26] | Knowledge, Attitude, and Practice Towards Reproductive Health Issues of Adolescents in Rural Areas, Indonesia: A Cross-sectional Study | 121 students high school students in the rural | Lack of Knowledge, The Role of Parents |
| Abraham et al., (2022) Southwest Ethiopia [39] | Rural Adolescents: Parental Communication on Sexual and Reproductive Health Matters in Jimma Zone, Southwest Ethiopia | 833 adolescents participated | Lack of Knowledge, The Role of Parents, Social and Cultural Norms, Difficult Access to SRH Services |
| Jisso et al., (2022) Ethiopia [40] | Sexual and reproductive health service utilization of young girls in rural Ethiopia: What are the roles of health extension workers? Community-based cross-sectional study | A total of 902 young girls were | The Role of Parents, Stigma and Discrimination, Difficult Access to SRH Services, Lack of Adequate Healthcare Professionals |
| Idowu et al., (2021) Nigeria [32] | Sexual Behaviour and Determinants of Reproductive Health Services Utilization Among Young People in a Rural Nigerian Community | 430 study participants | Lack of Knowledge, Feelings of Shame and Fear, Peer Relationships |
| Habte & Dessu, (2021) Ethiopia [23] | Uptake of Sexual and Reproductive Health Services and Associated Factors Among Rural Adolescents in Southern Ethiopia, 2020 | 1028 adolescents | Lack of Knowledge, Peer Relationships, Difficult Access to SRH Services, Lack of Adequate Healthcare Professionals |
